# Survey of Procedural Methods for Two-Dimensional Texture Generation

**DOI:** 10.3390/s20041135

**Published:** 2020-02-19

**Authors:** Junyu Dong, Jun Liu, Kang Yao, Mike Chantler, Lin Qi, Hui Yu, Muwei Jian

**Affiliations:** 1 Department of Computer Science and Technology, Ocean University of China, 238 Songling Road, Qingdao 266100, China; dongjunyu@ouc.edu.cn (J.D.); qilin@ouc.edu.cn (L.Q.); 2 Science and Information College, Qingdao Agricultural University, 700 Changcheng Road, Qingdao 266109, China; 3 Shandong Provincial Key Laboratory of Software Engineering, Shandong University, Jinan 250100, China; 4 Institution of Jinan Policy Research, Jinan 250001, China; yaokang@ouc.edu.cn; 5 Computer Science Department, Heriot-Watt University, Edinburgh EH14 4AS, UK; m.j.chantler@hw.ac.uk; 6 School of Creative Technologies, University of Portsmouth, Eldon Building, Winston Churchill Avenue, Portsmouth PO1 2DJ, UK; hui.yu@port.ac.uk; 7 School of Computer Science and Technology, Shandong University of Finance and Economics, Jinan 250014, China; 20173016@sdufe.edu.cn

**Keywords:** texture, procedural texturing, texture generation, procedural noise, texture perception

## Abstract

Textures are the most important element for simulating real-world scenes and providing realistic and immersive sensations in many applications. Procedural textures can simulate a broad variety of surface textures, which is helpful for the design and development of new sensors. Procedural texture generation is the process of creating textures using mathematical models. The input to these models can be a set of parameters, random values generated by noise functions, or existing texture images, which may be further processed or combined to generate new textures. Many methods for procedural texture generation have been proposed, but there has been no comprehensive survey or comparison of them yet. In this paper, we present a review of different procedural texture generation methods, according to the characteristics of the generated textures. We divide the different generation methods into two categories: structured texture and unstructured texture generation methods. Example textures are generated using these methods with varying parameter values. Furthermore, we survey post-processing methods based on the filtering and combination of different generation models. We also present a taxonomy of different models, according to the mathematical functions and texture samples they can produce. Finally, a psychophysical experiment is designed to identify the perceptual features of the example textures. Finally, an analysis of the results illustrates the strengths and weaknesses of these methods.

## 1. Introduction

Texture, as a basic property of the surface of an object, is an extremely important feature for describing and identifying objects which exist widely in nature. Tactile sensing and texture recognition are key components in the areas of aeronautical engineering, optical engineering, and many cyber-physical systems, as they can provide rich details regarding object surfaces. Novel sensor devices have been designed to improve tactile sensing, haptic perception, and surface texture recognition [1,2]. Accordingly, accurate and reliable methods for measuring the perceptual texture features of surfaces (e.g., roughness, fineness, and granularity) are in high demand, which can work together with sensors to provide integrated systems. In this context, a broad variety of textures that can simulate real surfaces are helpful in the design and development of new sensors.

Procedural models are essential for providing provide realistic surface details in simulation or animation systems [3]. They have been widely used in many research and application fields involved with cyber-physical systems (CPS), such as Virtual Reality and Augmented Reality. Such systems require a large amount of high-resolution textures to provide an immersive sensation for users. Such high-resolution textures may even be required to be generated in real-time [4,5].

A procedural texture is a computer-generated image created using a mathematical algorithm [3], which can be used for such tasks. The realistic texture images generated by procedural models are usually used to simulate surfaces of certain materials, such as wood, stone, and cloth, or extended to the textures of natural phenomena, such as clouds, steam, smoke, fire, water, and landscapes.

One of the advantages of using a procedural model to generate a texture is that they can generate textures with no limitation in size or resolution. It can provide a fully detailed texture no matter how high the resolution and cover an arbitrarily large area without seams or unwanted repetition of the texture pattern. More importantly, it can efficiently produce a class of related textures by varying the set of input parameters, rather than being limited to one fixed texture.

However, compared to other methods for acquiring texture images, procedural textures can be difficult to build. It is hard to predict the texture appearance when choosing a set of input parameters. A slight change in the value of one or more of the parameters may result in two texture images with completely different appearances. Even for experienced professionals, it is difficult to control the parameters to obtain a desired texture, not to mention for ordinary users.

Over the years, a large number of procedural texture models have been proposed or improved. However, procedural texture models are diverse and complex, and researchers from different areas (i.e., other than those in computer graphics) may not be familiar with them or even know about them. In recent years, procedural models have been receiving increased attention in the research community. Therefore, a comprehensive overview is necessary, especially for the benefit of researchers in other areas.

The objective of this survey is to provide readers with understanding and knowledge of several procedural texture models for creating realistic textures, such as marble, wood, stone, and other natural materials. Readers concerned with the areas of sensors and CPS research may be interested in procedural textures that can simulate real surfaces, which is helpful in the design and development of new sensors. Thus, in this survey, we limit the content to the generation of two-dimensional static procedural textures and we will not consider procedural generation techniques for other types of content, such as solid textures, games, animation, and so on. For readers who wish to know more about procedural models, the book [3] presents a detailed overview of procedural approaches to texturing, modeling, shading, and animation.

There are several issues that researchers may be concerned with:

1. For each model, what types of texture it can produce;
2. In terms of the mathematical background or output textures, whether there any methods that are similar;
3. For human observers, how to describe the texture images generated by these methods using perceptual features; and
4. What the strengths and weaknesses of these models are.

The first question considers looking for the representative textures that each model can produce, based on variation of the input parameters. The answer to the second question considers texture taxonomy. The third question involves asking human users or observers to view texture images generated by these methods and mark relevant perceptual features through psychophysical experiments. The last question mainly involves the comparison of different methods. Obviously, a complete survey of these models which answers the four above questions is desirable and would be very important for researchers not only from computer graphics community, but also in computer vision and many other research communities.

**Figure 1:**
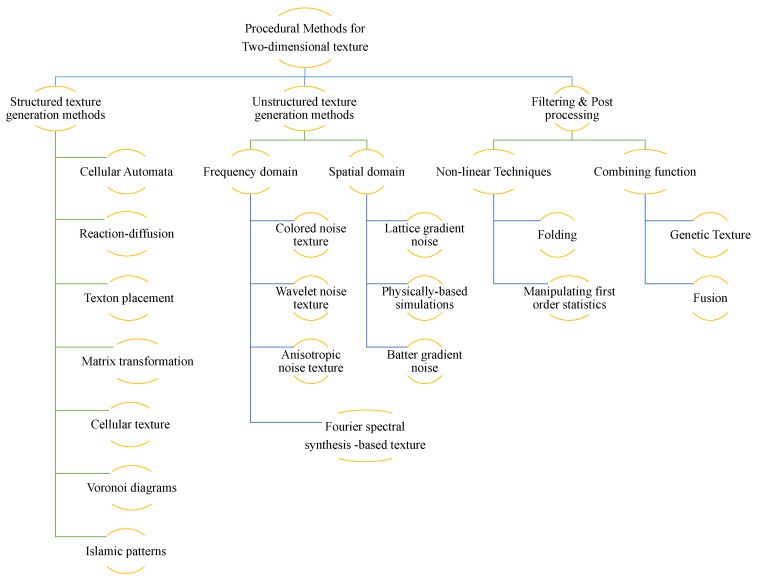
Procedural methods for two-dimensional textures.

In this survey, we aim to find answers to these four questions. We first split the available methods for generating procedural textures into two types: the first type is called generation methods, referring to those methods or algorithms that directly generate new texture images; the second involves filtering and post-processing to generate texture images. Relating to the first type (i.e., generation methods), we further divide it into two categories. Differing from other surveys, we have not categorized them according to the principle of the procedural method, but instead adopted a more intuitive way, classifying the methods according to human perception of the procedural textures; that is, the appearance of the texture that the model can produce. Some of the methods can produce textures with structured patterns and some appearance of regularity, while others can produce irregular textures with irregular primitive. Figure 1 illustrates the categories of procedural models.

We briefly introduce each method together with texture examples with varied appearances, which the method can produce by changing the input parameters. Moreover, using previous surveys related to texture generation [6,7,8,9] along with 2D static texture examples, we also show surface height maps and their rendered results produced by these methods. We are particularly interested in those methods that can generate rough surface textures which appear similar to real-world surfaces. Finally, we describe the characteristics of these models, based on the results of psychophysical experiments.

## 2. Structured Texture Generation Functions

We first introduce the available procedural methods which can directly generate new texture images. With respect to the textures generated by the procedural models, we considered whether the result texture is structured or unstructured. The pattern generated defines the texture pattern and sets the properties of the surface. A structured texture has a structured pattern with some appearance of regularity, while an unstructured texture has irregular primitives with the appearance of randomness.

A structured texture is defined not only by the shape and position of texture elements (called textons), but also the way that the textons are arranged with respect to each other.

### 2.1. Cellular Automata (CA)

A Turing pattern is a spatially heterogeneous pattern caused by diffusion, due to its instability. Cellular Automata and Reaction Diffusion (RD) are two models that generate Turing patterns.

A cellular automata is a discrete model that consists of a regular grid of cells, each in one of a finite number of states. The grid can be in any finite number of dimensions.

For each cell, its neighborhood is defined relative to the specified cell. An initial state (at time t=0) is selected by assigning a state for each cell. A new generation is created by advancing t by 1 and determining the new state of each cell, in terms of the current state of the cell and the states of the cells in its neighborhood, according to a certain rule (or rules). This rule is then applied iteratively for as many steps as desired. Wolfram proposed a numbering scheme for one-dimensional CA, which was called elementary cellular automata [10,11]. The local rules are described by an eight-digit binary number, where there are a total of 256 possible distinct CA rules in one dimension with a three-site neighborhood [12].

In two dimensions, the best-known cellular automaton is Conway’s game of life, proposed by Conway and popularized in Martin Gardner’s Scientific American columns [13]. In the literature [14,15,16,17,18,19,20], many other variations of cellular automata have been proposed.

When generating a texture, one may start from an array of differently-colored cells and then apply update rules to determine the colors of the next array of cells.

A good property of cellular automata is that, even with the simplest update rule based on two colors (e.g., if the current cell is the first color and the cells to the right and left are the second color, then the next cell in the evolution shall be the first color), can produce a complex texture. Below, we briefly describe the procedure to generate an example of a cellular automaton called the forest fire model:

1. Define three different states for each cell: state = 0 is empty, state = 1 is burning and state = 2 is forest;
2. Check one or more of the four neighbors of a cell. If there is one neighbor whose state is burning (state = 1) or forest (state = 2), then the new state is burning (state = 1);
3. Assign a low probability (e.g., 0.000005) for a forest cell (state = 2) to start to burn on its own (i.e., from lightning);
4. Change a cell that is burning (state = 1) to empty (state = 0);
5. Assign a low probability (e.g., 0.01) to an empty cell becoming forest (i.e., to simulate growth);
6. Connect the array, such that fire which burns all the way to the left side will start fires on the right side. Similarly, connect the top and bottom.

**Figure 2:**
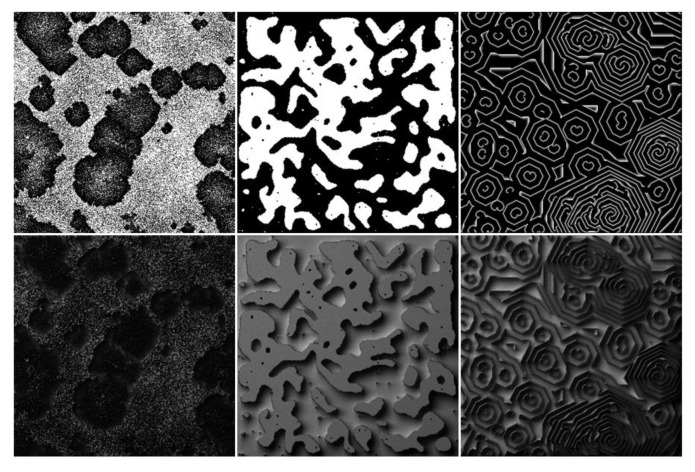
Textures generated with cellular automata. Upper left: Texture generated with the forest fire model. Bottom left: Relighting result. Upper middle: Texture generated with the surface tension model. Bottom middle: Relighting result. Upper right: Texture generated with the excitable media model. Bottom right: Relighting result.

Figure 2 shows example images produced by cellular automata.

### 2.2. Reaction–Diffusion Algorithm (RD)

Reaction–diffusion was first introduced by Alan Turning in 1952 as a chemical mechanism for pattern formation. It is a process in which two or more chemicals diffuse at unequal rates over a surface and react with one another to form stable patterns, such as spots and stripes. Texture generation algorithms can be derived by simulating this mechanism, by means of solving partial derivative equations. The key feature is that a small amount of variation in the initial concentrations gives an initially unstable system, which may then be driven into a stable state. The chemical concentrations in this stage vary across the surface and produce a pattern [21,22,23,24]. Several variations of reaction–diffusion algorithms are available, such as Chequer-board, Gray–Scott, and Swift–Hohenberg. The difficulty in using reaction–diffusion algorithms for texture generation is that small variations in the input parameters may produce dramatically different textures.

#### 2.2.1. Gray–Scott

The Gray–Scott model is a popular reaction–diffusion texture generation algorithm, which can produce a large variety of patterns with a biological appearance [25]. Some patterns may look like cell division, gastrulation, or the formation of spots and stripes on furry animals. The partial differential equation given by Equations (1) and (2), which model this process, may be solved by different numerical techniques:

(1) $$\partial u/\partial t=r_{u}{△}^{2}u-uv^{2}+f\left(1-u\right),$$

(2) $$\partial v/\partial t=r_{v}{▽}^{2}v-uv^{2}+\left(f+k\right)v.$$

Chemical reaction:

(3) U+2V→3V,

(4) V→P,

where *U*, *V*, and *P* are chemical species; *u* and *v* represent their concentrations; $r_{u}$ and $r_{v}$ are their diffusion rates; *k* represents the rate of conversion of *V* to *P*; and *f* represents the rate of the process that feeds *U* and drains *U*, *V*, and *P*.

As with all RD models, these patterns are the result of an iterative process which evaluates each cell of the simulation space based on the concentrations of the two main parameters (for Gray–Scott, these are usually named *f* and *k*) of the reaction equation. The constraint is the concentrations of these two substances in neighboring cells. Diffusion may be modeled by an explicitly conservative exchange process among neighbors, where the reactions are locally modeled at each locus by a simulated processor.

#### 2.2.2. Swift–Hohenberg Equation

**Figure 3:**
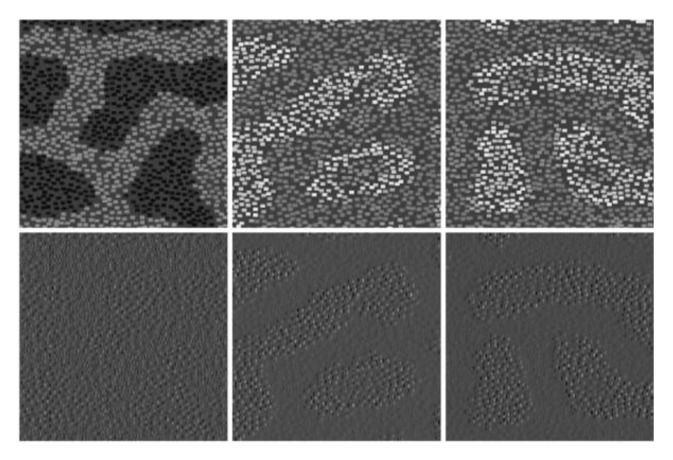
Textures generated with the Gray–Scott Reaction–Diffusion (RD) algorithm. First, row: height maps. Second row: relighting results.

The Swift–Hohenberg equation is a partial differential equation that can generate various patterns; the model is:

(5) $$\partial u/\partial t=ru-{\left(1+{▽}^{2}\right)}^{2}u+N\left(u\right),$$

where u=u(x,t) or u=u(x,y,t) is a scalar function defined on the line or plane, respectively; *r* is a real bifurcation parameter; and N(u) is some smooth nonlinearity. The equation is named after the authors of the paper [26], in which it was derived from the equations for thermal convection. The evolution of random initial states under the Swift–Hohenberg equation exhibits two stages of relaxation. The initial phase can be described by power law decay; in this stage, local striped domains emerge from a noisy background. Slower power law decay can lead to coarsening of the striped domains. Transition between the phases is achieved due to different time scaling, leading to the collapse of distinct curves. Figure 3 shows example textures generated using the Gray–Scott RD model.

#### 2.2.3. Discussion

CA models provide an alternative way to generate Turing Pattern by solving Reaction–Diffusion PDEs [16,27]. In [27], Adamatzky et al. employed the beehive hexagonal cellular automaton to design a discrete model for a chemical reaction–diffusion system. Three species—substrate, activator, and inhibitor—are involved in this system. As an example, a compact pattern generator (or a glider gun), which is essential for implementing negation, was provided in their paper. Therefore, reaction–diffusion and hexagonal cellular automata are logically universal, which allows for the embedding of logical circuits and can potentially implement meaningful computational operations. In [16], a CA algorithm has been used to simulate and investigate reaction–diffusion systems. This method provides a way to investigate and analyze spatio-temporal dynamics, especially in Turing pattern formation. CA models are discrete models involving parameters in space, time, and state, and differ from PDEs. The reaction and diffusion process can be simulated by introducing different evolution rules with the help of parameters in the CA model (i.e., the lattice *C*, the state set *I*, and the interaction neighborhood *N*). The authors conducted simulations for the Brusselator problem and the Gray–Scott problem. They concluded that the ability of CA models to qualitatively capture the solution behaviors of reaction–diffusion PDEs shows sufficient promise.

### 2.3. Texton Placement

Texton placement algorithms produce textures by placing different textons, which are normally simple geometry elements defined by a few parameters [28,29,30]. While changing these parameters can produce visually different textures, the rule for placement is also important for generation.

#### 2.3.1. Placement Rules

There are a number of rules that can be used to control the way a texture is produced. The section only describes the most commonly used ones. Common rules include scattering “feature points” through $R^{3}$ (i.e., three-dimensional space) and building a scalar function based on the distribution of the local points. There exist several algorithms; for example, “bombing” is a technique which places geometric features such as ellipsoids throughout space, which generate patterns on surfaces that cut through these features.

##### Regular Grid

This is the most similar method to simply generating a regular texture. Basically, a regular distribution of placements (e.g., a Cartesian grid) is used.

Here, we used ellipsoids placed using a regular grid to generate a texture [31]. Suppose the length of the semi-major axis is *a*, the length of semi-minor axis is *b*, and the height of ellipsoid is *c*. Then, textures can be generated as follows:

1. Create geometric feature (i.e., ellipsoids):
  (6) $$pixel=\sqrt{c^{2}\left(1-\left(row^{2}/a^{2}+col^{2}/b^{2}\right)\right)}$$
  row and col are coordinates in *c*; −a≤row≤a, −b≤col≤b;
2. Divide the space into a grid of uniformly spaced cubes. The locations of these cubes must be fixed according to the order of the grid, which is regular. It should be noted that the size of a cube must be the same as the size of the ellipsoid defined in the same step.
3. Place the ellipsoids generated in the first step into the spaced cubes, and then change the values of a,b,c. A number of iterations will produce visually different textures. The iteration number is decided by the number of ellipsoids.

##### Random Grid

This method is essentially the same as regular grid placement method, except that randomness is added into the *x*–*y* vector at each point (placement) in the regular grid. For example, we may use vectors drawn from a 2D Gaussian distribution.

##### Random Walk

This is an algorithm that basically places any textons in a random manner. It is slightly different from the previous algorithm and can be described as follows:

1. Randomly initialize a location ($x_{1},y_{1}$) in the image and place a texton, as defined in Equation (6);
2. Add a random vector drawn from a 2D Gaussian to ($x_{1},y_{1}$) to produce the $2^{nd}$ point ($x_{1},y_{1}$). Then, another texton is placed at ($x_{1},y_{1}$);
3. Repeat Label (2) for a number of steps.

When the parameters (i.e., a,b,c) of the basic texton and the number of iterations are changed, visually different textures will be produced.

##### Probability Map

This algorithm uses a height-map to control the probability of texton placement. The procedure is as follows:

1. Generate a fractal height map;
2. Randomly generate a point (x,y);
3. Look (x,y) up in the height-map;
4. If the height at (x,y) is greater than the threshold, then place a texton as defined in Equation (6). Otherwise, nothing will be changed;
5. Repeat Steps 2–4 for as many placements as one wishes.

**Figure 4:**
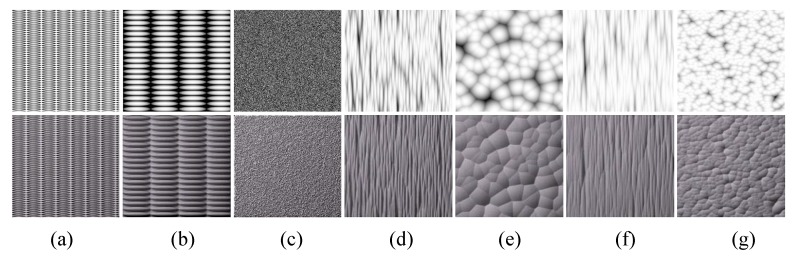
Textures generated by ellipsoids placed using different placement rules: (**a**,**b**) are samples generated by a regular grid rule; (**c**) is generated by a random grid rule; (**d**,**e**) are generated by a random walk rule; and (**f**,**g**) are generated by a probability map rule. Samples in the first row are height maps and samples in the second row are relighting results.

Figure 4 shows some textures generated by using ellipsoids with different rotation angles and different combination rules.

#### 2.3.2. Texton Generation

The previous methods all used ellipsoids parameterized by the semi-major axis *a*, the semi-minor axis *b*, and the height of ellipsoid *c*. A parameter that defines the orientation of the ellipsoids was also used. We may vary these parameters by sampling them from a probability map (e.g., they may be generated by a Gaussian distribution).

These images are almost the same as those produced by the random placement rule; the difference is that the ellipsoids in the cube are generated with clockwise or anti-clockwise rotations. In this way, we can generate texture images with various orientations.

#### 2.3.3. Texton Combination

**Figure 5:**
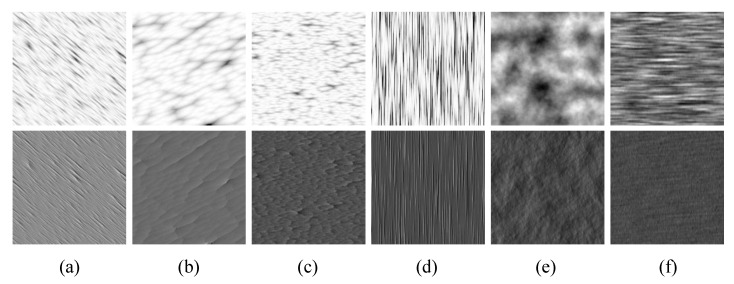
Textures generated using ellipsoids with different rotation angles and different combination rules: (**a**,**b**) are samples generated by using ellipsoids with different rotation angles; (**c**,**d**) are generated by a max rule; and (**e**,**f**) are generated by an addition rule. The samples in the first row are height maps and the samples in second row are relighting results.

Various textures can also be generated by combining different textons. Many texton combination rules can be used. We list only the simple and typical ones below. Figure 5 demonstrates the textures generated using ellipsoids placed with different placement rules.

1. The maximum rule:
  Basically, the maximum value is used when there are overlapping textons at a certain location (i.e., the pixel value is taken from the maximum value at the same location).
2. The addition rule:
  The placement rule for ellipsoids is the same as those listed in the previous section, but the difference is that the ellipsoids are placed in 3D space. In other words, at a certain point (x,y), the value is taken from the addition of two ellipsoids when they are overlapped, instead of being replaced by the maximum of the two.

### 2.4. Matrix Transformation

Matrix transformation is a simple method for generating textures [32]. A source image is used, and pixel positions are changed using a matrix transformation as many times as the user wishes; however, the elements of the matrix must be Lucas or Fibonacci. This method is able to generate a large amount of different textures rapidly and is especially capable of producing various textures which simulate real fabrics.

The procedure is as follows. First, a source image is required; it can be any kind of image and it does not have to be very complicated. Even a simple source image can generate good results. Next, an appropriate transformation matrix needs to be selected to transform the location (x,y) in the source image to ($x^{\prime },y^{\prime }$), which is done using Equation (7):

(7) $$\left[\begin{array}{c} x^{\prime }\\ y^{\prime } \end{array}\right]=\left[\begin{array}{cc} a_{11} & a_{12}\\ a_{21} & a_{22} \end{array}\right].\left[\begin{array}{c} x\\ y \end{array}\right].\;(\mathrm{mod}\;N),$$

where $a_{12}=a_{21}=0$, and $a_{11}$ and $a_{22}$ are Lucas or Fibonacci; that is, $a_{11},a_{22}\in \left\{1,3,4,7,11,18,29,47,76,123,199,\cdots \right\}$ or $\in \left\{1,1,2,3,5,8,13,21,34,55,89,144,233,\cdots \right\}$.

In addition, the product of $a_{11},a_{22}$ and 256 must be prime to each other. As the number of iterations increases, we can obtain textures with various appearances.

**Figure 6:**
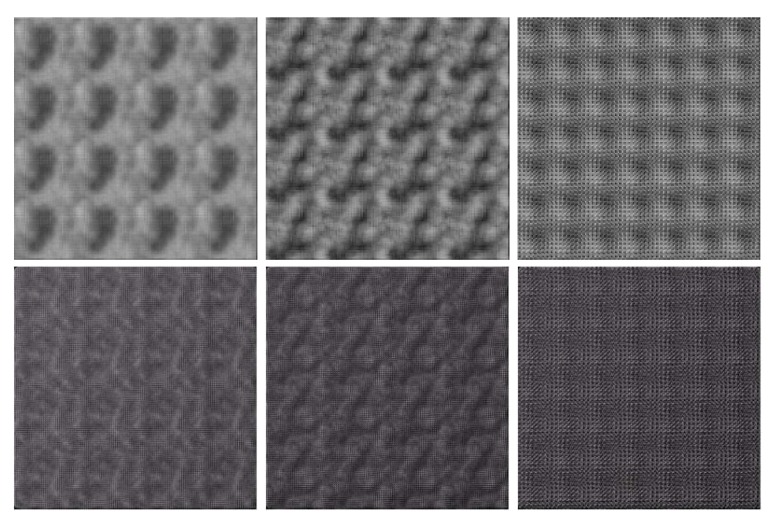
Matrix transformation textures. First, row: height maps. Second row: relighting results.

The texton placement and matrix transformation methods are both based on the placement of textons or pixels. In particular, the matrix transformation-based method moves pixels to different locations through transformation using different operators (i.e., matrices). Thus, the pixels are treated as textons in this case. Although the appearance of the textures generated by the two methods may look different, they are fundamentally similar in terms of theory and implementation. Figure 6 shows samples generated using matrix transformations.

### 2.5. Cellular Texture

The basic idea of a cellular texture is that a set of points is first generated in a certain way and scattered randomly throughout the image, and then, for each pixel, its distance to the nearest couple of points is calculated; those values are normalized to determine the color.

We briefly explain a typical method for cellular texture generation by Worley, who presented a new basis function, which complements Perlin noise by partitioning space into a random array of cells [33]. This basis function can be used to produce textured surfaces resembling flagstone-like tiled areas, organic crusty skin, crumpled paper, ice, rocks, mountains, and craters. The basis function was designed from the idea of random feature points. Image points are randomly distributed through all of $R^{3}$. For any location *x*, there are some feature points which lie closer it to than any other feature point. Define $F_{n}\left(x\right)$ as the distance from one random point to the $n_{th}$-nearest feature point.

The properties of the function *F* include:

1. $F_{n}$ is always continuous, and
2. $F_{n}$ is nondecreasing: $0\leq F_{1}\left(x\right)\leq F_{2}\left(x\right)\leq F_{3}\left(x\right)$.

In general, $F_{n}\left(x\right)\leq F_{n}\left(x+1\right)$ by definition of $F_{n}$. The gradient of $F_{n}$ is simply the unit direction vector from the $n_{th}$ closest feature point to *x*.

In order to compute the functions $F_{n}$, Worley’s approach divides space into a grid of uniformly spaced cubes, separated at the integer co-ordinate locations. Each “cube” in space can be uniquely represented by its integer coordinates and determined by simple floor operations. As we generate *m* random feature points, we compute the distance to the original function evaluation location *x*, and keep a sorted list of the *n* shortest distances we have obtained so far. The $n_{th}$ shortest distance is $F_{n}\left(x\right)$. Thus, we can effectively find values for $F_{1},F_{2},F_{3},\cdots ,F_{n}$ simultaneously.

The above method is effective for the generation of a solid texturing primitive. As with the Perlin noise function, mapping the values of this function into a high color value can produce visually interesting and impressive effects. There are many combinations, such as: $F_{1}-F_{2},F_{1}+F_{2},2F_{3}-F_{2}-F_{1}$.

**Figure 7:**
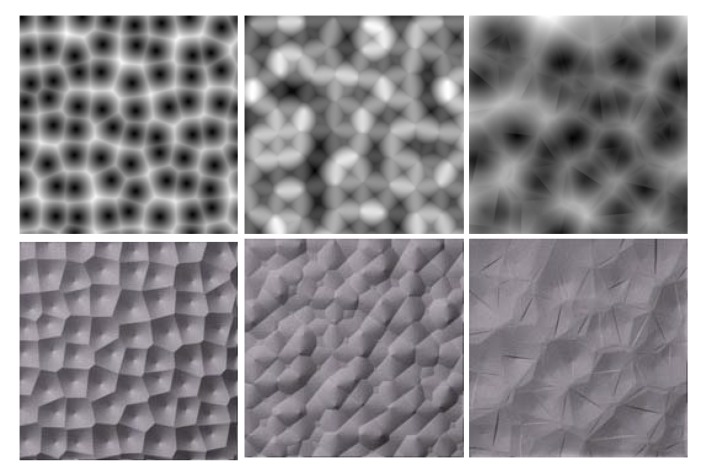
Textures generated by cellular algorithms. First, row: height maps. Second row: relighting results.

It can be seen that $F_{4}$ and other higher *n* start producing similar textures, but the lower values of *n* (up to 4) may have a distinct effect. Therefore, we recommend using combinations of these low-*n* basis functions $C_{1}F_{1}+C_{2}F_{2}+C_{3}F_{3}+C_{4}F_{4}$ for various values of $C_{n}$. Figure 7 shows textures generated by cellular algorithms.

### 2.6. Voronoi Diagrams

This section introduces methods that are explicitly related to geometric operations to generate textures. Again, we are particularly interested in those methods that can produce textures resembling natural ones.

Voronoi diagrams have been demonstrated as a method for procedural texture generation in [33], in which the authors detailed an algorithm that partitions space into a random array of cells to create cellular-looking textures. Voronoi diagrams have been widely used in many fields [34,35,36]. For procedural texture generation, suppose we have a set of points *S* in the plane (which are called Voronoi sites). Each point has a Voronoi cell V(s) which consists of all points closer to s than to any other site. The segments of the Voronoi diagram are all the points in the plane which are equidistant to the two nearest points. The Voronoi nodes are the points equidistant to three (or more) sites. The procedure for generating Voronoi diagrams is as follows:

1. Generate the initial point set $\left\{z_{i}\right\}$ composed of *k* points;
2. Initialize the Voronoi dipartite region $\left\{v_{i}\right\}$ in the current region *R*;
3. In region $\left\{v_{i}\right\}$, compute the center points, and then set these center points as the new point set $\left\{z_{i}\right\}$;
4. If points in the new point set satisfy the convergence condition, the algorithm stops; otherwise, go to (2) and repeat (2)–(4).

**Figure 8:**
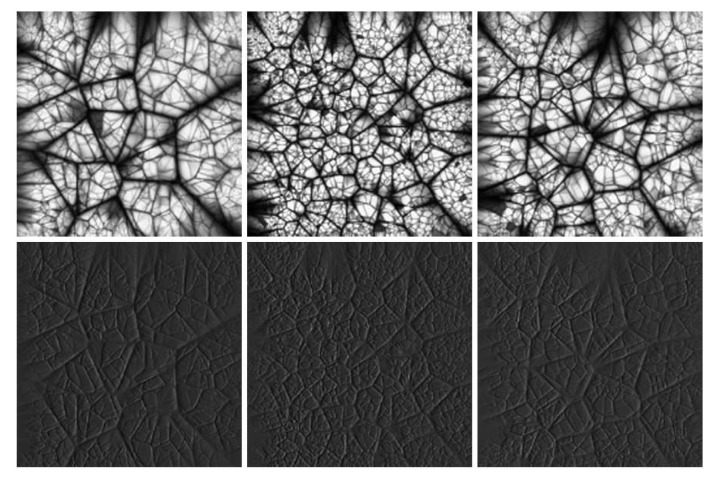
Textures generated using the Voronoi Diagram algorithm. First, row: height maps. Second row: relighting results.

Variations of the traditional Voronoi diagram have been demonstrated. For example, one may also input a grid or a randomized grid pattern of dots to initialize the process. Figure 8 shows examples of Voronoi textures.

### 2.7. Islamic Patterns

Islamic patterns refer to the patterns that can be found in the Islamic world. These designs have existed for more than a thousand years. The basic idea in forming an Islamic pattern is to construct intricate geometric art based on radially symmetric star-like figures.

**Figure 9:**
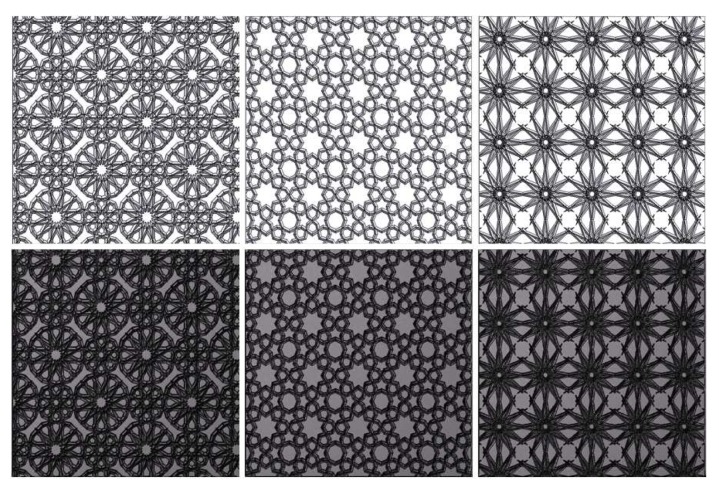
Islamic pattern textures. First, row: height maps. Second row: relighting results.

In [37,38,39,40], several methods have been proposed for the generation of textures using Islamic patterns. The basic idea is to first construct a plane with some regular polygons, which are then filled with radially symmetric motifs like those found in the Islamic tradition. For example, star polygons and rosettes are commonly used as motifs. An *n*-pointed star polygon can be generated in the following way: first, *n* points are evenly sampled along the perimeter of a circle. Then, a number *d* with a value smaller than *n* is chosen; finally, each point is connected to the $d_{th}$ point encountered after it on the circle. A rosette is essentially a star, but where hexagons are placed in the concavities between its adjacent points. In order to generate an Islamic pattern, *n-gon* polygons can be used to tile a plane. For each regular *n-gon*, an *n-fold* star, rosette, or extended rosette can be placed into it. Gaps between the regular polygons are then filled by finding natural extensions of the lines meeting their boundaries. The final texture exhibits graph-theoretic properties, with lines of various lengths and thicknesses. Colors may also be used in various ways (e.g., interlacing to make the pattern similar to a weave). Figure 9 shows some examples of Islamic patterns.

Cellular textures, Voronoi textures, and Islamic textures can be treated as belonging to the same class, as they are all based on Thiessen polygons, which are polygons whose boundaries define the area that is closest to each point relative to all other points. The seed point set used in a Voronoi Diagram exactly corresponds to that in a cellular texture, in which a pixel value is generated based on the combination of distances to some seed points. These methods are all explicitly related to geometric operations to generate a texture.

## 3. Unstructured Texture Generation Functions

Noise functions are the most commonly used means for unstructured procedural texture generation. The authors in [3] outlined some recent methods for noise texture function generation. Although this type of texture does not focus on the problems this paper is mainly concerned with, we would still like to list some representative methods. Differing from [3], we divide noise generation functions into two types of methods, frequency domain and spatial domain approaches, such that readers can view these functions from a different angle.

### 3.1. Frequency Domain

A typical unstructured texture can be seen as noise, which is normally a stationary and random process, and can be efficiently generated in the frequency domain. Pixel values in a texture image can be obtained by specifying the amplitude and phase for every frequency and then taking the inverse Fourier Transform. For an unstructured texture, the phase spectrum can be random. As it is generally believed that humans have difficulty in distinguishing images that differ only in higher than second-order statistics, the autocorrelation (or power spectrum), which can represent the second-order moment, has been commonly used for noise texture generation. A random phase is often added to spectrum generated by certain functions, following which the resulting texture will be unstructured. Moreover, the power spectrum is physically motivated and is a familiar concept; it is usually used as a representation of the noise. In this way, we can secure an expected noise texture simply by controlling the power spectrum.

#### 3.1.1. Colored Noise Texture

In signal processing, a colored noise process is a random process whose power spectral density (PSD) is not white or nearly white. White noise is named in analogy to white light, which has a flat frequency spectrum. Thus, a white noise signal has equal power in any band of a given bandwidth (power spectral density). The “color” terminology is here derived from the classification by spectral density. There are also different names, based on colors, for different spectral densities. In contrast to white noise, colored noise does not vary completely randomly. They can be generated from white noise by passing it through a low-pass filter; such a system is called a shaping filter, and the appearance of a colored noise texture can be changed by adjusting the parameters of the shaping filter.

#### 3.1.2. Wavelet Noise Texture

Although the original Perlin noise function is simple and has been widely used to generate procedural textures [41], it is prone to problems with aliasing and detail loss. Wavelet noise has been designed to reduce this problem by employing a wavelet transform. Thus, we classify wavelet noise as a frequency domain approach. The basic idea of wavelet noise is as follows [42]:

1. An image *R* filled with random noise is created;
2. Downsample *R* to create a half-size image R↓;
3. R↓ is upsampled to a full-size image R↓↑;
4. Subtract R↓↑ from the original image *R* to create *N*;
5. *N* is then used in the same way as Perlin noise to construct noise patterns.

**Figure 10:**
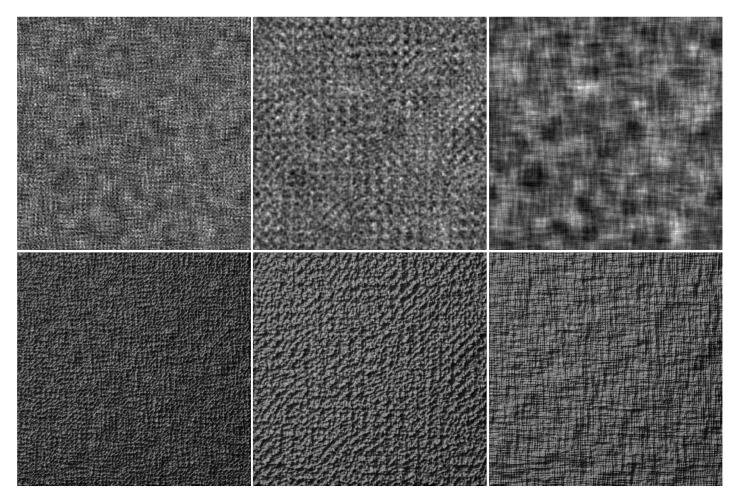
Textures generated with wavelet noise. First, row: height maps. Second row: relighting results.

The construction of *N* is similar to the procedural band-pass pyramids of [43]. We use a wavelet noise function to generate *3D* surface height maps and then relight them. Parameters are varied, and we are able to generate surfaces with complete randomness and strong directionality. Figure 10 shows texture samples generated with wavelet noise.

#### 3.1.3. Anisotropic Noise Texture

Similar to wavelet noise, anisotropic noise was originally proposed to generate high-quality noise textures for interactive applications [44]. The goal also includes reducing the aliasing effect. The basic idea is to use oriented and narrowly band-limited sub-bands to generate a set of images; the final texture is obtained as a weighted sum of these images. The generation method is based on steerable filters, similarly to wavelet noise [45,46]. Texture tiles are pre-computed, instead of sampling noise procedurally. However, the frequency content of the tiles is strongly oriented and anisotropic. Thus, anisotropic filtering may be performed during rendering.

#### 3.1.4. Texture Generation Based on Fourier Spectral Synthesis

Fourier spectral synthesis is one of the most commonly used techniques to generate random textures. The fundamental theory is to generate a noise texture with a specific power spectrum by filtering white noise in the frequency domain. A typical class of textures produced using Fourier spectral synthesis is fractals, which can simulate many natural phenomena, such as rock surfaces and sand ripples.

##### Fractal Models

Fractal models have been commonly used in many graphical applications, as they can generate natural textures with self-similar structures at different scales. The archetypal fractal procedural model for generating textures is called fractional Brownian motion (fBm). Fractals can be constructed from literally any basis function; typical basis functions for fBm are Perlin noise functions or Voronoi functions [3,47,48].

We use one-over-fBeta-noise (random phase fractals) as an example, which has been widely used to generate textured surfaces with relief features (e.g., rocks and terrain):

(8) $$M\left(f,\theta \right)=\left(1/f^{\beta }\right)\left(e^{-{\left(\theta -\theta_{0}\right)}^{2}/2\sigma^{2}}\right)\left(\frac{\delta }{\delta_{n}}\right),$$

where M(f,θ) is the polar representation of the magnitude spectrum, where *f* is the radial frequency and θ is the angular frequency; $\theta_{0}$ is the dominant angular frequency and $\delta_{n}$ is the RMS roughness normalization factor; β is the roll-off factor; δ is the RMS roughness; and $\sigma^{2}$ is the angular variance.

**Figure 11:**
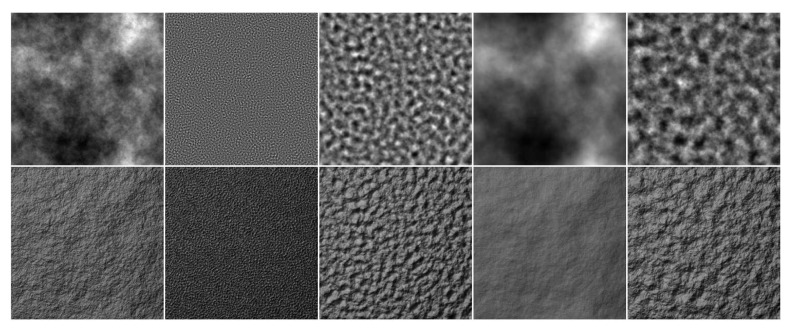
Textures generated with fractal models. First, row: height maps. Second row: relighting results.

By simply combining a suitable power spectrum function and random phase, many rough surfaces resembling natural textures can be produced. Figure 11 shows some fractal texture samples. There are also various models which take different parameters as input and can generate different types of textures.

##### Sparse Convolution Noise Texture

Sparse convolution is another type of noise texture generation method based on Fourier Spectral Synthesis. The sparse convolution noise is synthesized by the convolution of a three-dimensional kernel and a Poisson noise process [49]. The Poisson impulse process consists of impulses of uncorrelated intensity at uncorrelated locations. As there are only a few scattered impulses in the process and it has a constant power spectrum, the process is called sparse white noise.

The steps in generating a kernel are as follows:

1. Generate white noise;
2. Filter the noise to produce the desired spectral result;
3. Transform the signal back to the spatial domain; and
4. Window the result to produce the kernel to be used for convolution.

The advantage of this techinique is that it can provide improved spectral control and allow a trade-off between quality and efficiency without introducing gross artifacts.

#### 3.1.5. Spot Noise

Spot Noise is a texture synthesis technique that can be used for vector field visualization [50]. A spot noise texture can be generated by blending together a large number of small intensity functions at random positions on a plane. For visualization, the vector field determines the shape of the intensity functions, which are also called spot functions. In the frequency domain, spot noise is generated by multiplying the Fourier Transform of a spot function with a scale factor and a random phase shift. Thus, the spot noise method can be seen as a frequency domain method. Recently, a spot noise method based on a controlled distribution of kernels, as an alternative formulation to local random phase noises aligned on a regular grid, has been introduced. This noise model improves control over local structural features, while keeping the benefits of local random phase noise [51,52].

#### 3.1.6. Gabor Noise

Gabor noise can be seen as an extension of sparse convolution noise, which was proposed in order to accurately control the spectrum using Gabor kernels [53,54,55]. In computer graphics applications, it has the advantage of requiring very little memory and producing non-periodic and anisotropic textures. The other advantage of Gabor noise is that it does not require texture parameterization when it is mapped onto a surface.

#### 3.1.7. Stochastic Subdivision

Stochastic subdivision [56] can be seen as another extension of fractal models. It can generate natural irregular fractal-like objects and phenomena. The original stochastic subdivision algorithm is derived from Fractional Brownian noise, which belongs to $1/f^{\beta }$ noise [57]. The recursive subdivision technique can be used in the context of stochastic modeling, and it has the advantage that the depth of the recursion may be adapted to the on-screen resolution. A generalized version of the stochastic subdivision technique, presented by Lewis, can control the autocorrelation and spectral properties of the synthesized random functions [58]. Natural results can be generated, as the model can produce a large amount of detail by specifying characteristic structural or statistical features.

### 3.2. Spatial Domain

There are many approaches to generate procedural textures in the spatial domain, among which the most famous one is Perlin noise [42]. We divide the spatial domain approaches into the following categories:

#### 3.2.1. Lattice Gradient Noise

**Figure 12:**
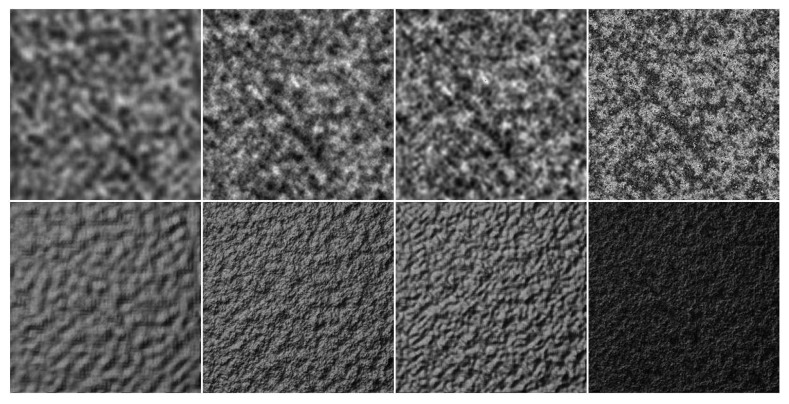
Textures generated with Perlin noise. First, row: height maps. Second row: relighting results.

The most famous and representative lattice gradient noise is Perlin noise, which generates a noise texture at a spatial location based on a pseudo-random gradient and interpolation [59]. Thus, a noise function and an interpolation function are essential to generate a Perlin noise texture. A noise function can be a simple seeded random number generator, which may take an integer as a parameter and return a random number based on that parameter. A Perlin noise function is typically composed of a combination of many different functions. These noise functions provide a random number calculated through one or more parameters. The output numbers are then smoothed, to make it less random-looking; many smoothing filters have been developed for this purpose. In the interpolation stage, standard interpolation functions can be used, such as linear interpolation, cosine interpolation, or cubic interpolation. Another two parameters are the amplitude and frequency when noise functions are added together. Perlin noise may be created by using functions with different characteristics and other frequencies and amplitudes ($frequency=2^{i},amplitude=persistence^{i}$) at each step. Each successive noise function added is known as an octave. Finally, all noise functions are added together to create the Perlin noise function. Some papers have presented noise functions based on other lattices (rather than the typical integer lattice) [60,61]. In [60], a lattice convolution noise based on a densely and evenly packed grid was proposed; and a Perlin-like noise (called simplex noise) was introduced in [61]. These approaches have the advantages of lower computational complexity and less directional artifacts. Figure 12 shows some Perlin noise textures.

#### 3.2.2. Physically-Based Simulations

Physically-based simulations refer to those procedural methods based on mathematical models for animating a turbulent fluid or similar natural phenomena. Compared to traditional solution using equations of fluid motion based on PDEs, these procedural methods have much smaller computational requirements and offer more animation control. These methods have also been called dynamic texture methods in the literature. Commonly used techniques in these methods include extensions and variations of Perlin noise. Fluid-like turbulent motion (e.g., smoke and vapor) are particularly suitable for generation using this type of technique.

##### Flow Noise

Flow noise can be seen as an extension of flow textures, which have been defined with shaders based on Perlin noise. However, they lack the swirling and advection effects of real fluid flow. Flow noise essentially extends Perlin noise by rendering over time to produce flow textures with a moving impression.

##### Curl-Noise

Curl-noise aims to generate realistic turbulent velocity fields with solid boundaries and controllable amplitudes. It is also based on Perlin noise and uses the curl of a potential field for velocity.

#### 3.2.3. Better Gradient Noise

Kensler et al. [62] proposed to improve the spectral properties of Perlin noise by relatively minor changes. Although the relevant discussion was based on frequency domain terminologies, the actual implementation was in the spatial domain. Three improvements were made to Perlin noise: a new hash function, a reconstructed kernel, and a reconstructed stencil projected onto a surface normal. In this way, better gradient noise texture without discernible periodicity, anisotropy, or aliasing can be produced.

## 4. Filtering and Post-Processing

In this section, we present the available methods that use filtering and other post-processing techniques to change an input texture, such that new textures can be generated. These methods differ from those introduced in the previous sections, as they do not generate textures directly from a mathematical model and an input texture is required. However, variation of parameters in these methods can produce dramatically different textures.

### 4.1. Nonlinear Techniques

#### 4.1.1. Folding

Folding operations can produce nonlinear results by reduction, accumulation, compression, and injection. It can be achieved by simply iterating an arbitrary function over a data structure in some order and generating a return value. Typically, a folding operation involves a combining function and a list of elements of some data structure. It proceeds to combine elements of the data structure using the function in some systematic way. The procedure of an example fold operation is as follows:

1. Select a source image *A*.
2. Scale the minimum of *A* as 0, fold_A=maxA/(nfolds+1), where maxA is the maximum of *A* after scaling, nfolds is number of folds; and fold_A is intermediate variable.
3. A=abs(A−fold_A); repeat (3) nfolds times.

**Figure 13:**
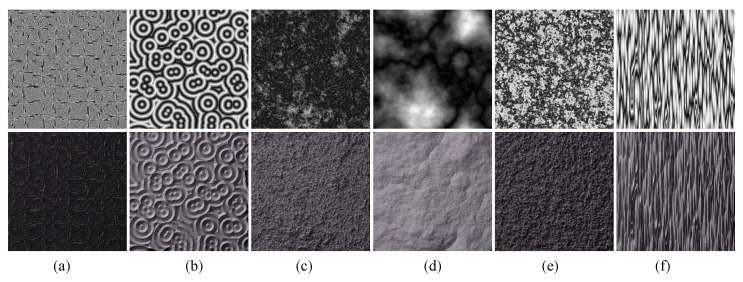
Textures generated by folding operations. First, row: height maps. Second row: relighting results. The source images of column (**a**,**b**) are cellular textures; the source images of column (**c**,**d**) are fractal textures; the source image of column (**e**) is a Perlin noise texture; and the source image of column (**f**) is a texton texture.

Figure 13 shows texture samples produced by folding operations.

#### 4.1.2. Manipulating First-Order Statistics

Some filtering and post-processing methods change the power density functions of a texture; for example, those basic transformation functions can change the image histogram. We classify this type of method as manipulating first-order statistics. Thus, any histogram modification algorithm would fall into this category. Different combinations of filters can also lead to varied first-order statistics while generating different textures.

**Figure 14:**
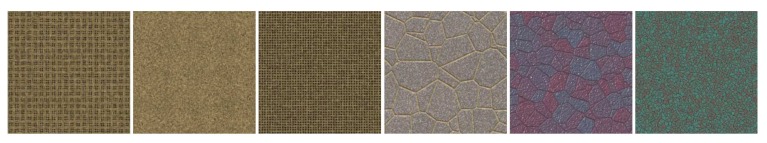
Example textures created by NeoTextureEdit.

Figure 14 shows example textures created by NeoTextureEdit, which is an open-source texture editing tool available from (http://neotextureedit.sourceforge.net/). Several filters, including blending, embossing, and warping, are used to create and mix different textures.

### 4.2. Combining Functions

This section introduces those methods that can combine the different functions introduced in previous sections. Obviously, there is a large number of possible functions.

#### 4.2.1. Genetic Texture

Genetic texture is an approach that employs a genetic algorithm to generate a new texture. The original Genetic Algorithm (GA) [63,64,65,66] was proposed by Holland [23] in 1975; it is a global stochastic optimum search algorithm based on the principles of genetics and biological evolution, in which a chromosome’s probability of being passed on to the next generation is determined by judging its ability to adapt to the environment. The key idea of a Genetic Algorithm is to eliminate low-fitness individuals while choosing high-fitness ones to do the genetic manipulation (i.e., crossover and mutation operations). The genetically manipulated individuals then form the next new generation. Thus, a GA can provide an approximate optimal solution to an optimization problem by iterative evolution.

A genetic texture generation algorithm can take a set of texture samples as an input and use some pre-defined function to completely modulate the eventual output textures. Users can pick a selection and the genetic algorithm then generates another set of textures by mutating and crossing over elements of the user-selected textures.

#### 4.2.2. Combination

Two different generation models can be combined by using wavelet decomposition and reconstruction [67]. The new algorithm can, then, generate various textures differing from the original input. Below, we show an example of a combination algorithm. A cellular texture can be generated by the basis function as described in Section 2.5. The texton placement algorithm is based on the ”bombing" technique, which places geometric features throughout space. As we are using ellipsoids in this example, the generated textures are named ellipsoids. The essence of our combination algorithm consists of the following steps:

1. Select two source images *A* (Cellular) and *B* (Ellipsoid);
2. Decompose *A* and *B* by respective wavelet transforms to create the decomposition coefficients $\left[\begin{array}{c} C_{A},S_{A} \end{array}\right]$ and $\left[\begin{array}{c} C_{A},S_{A} \end{array}\right]$;
3. Perform a weighted combination of the decompositions: $C=W_{1}C_{A}+W_{2}C_{B}$ and $S=W_{1}S_{A}+W_{2}S_{B}$;
4. Reconstruct *C* and *S* by a wavelet transform to create a new image.

**Figure 15:**
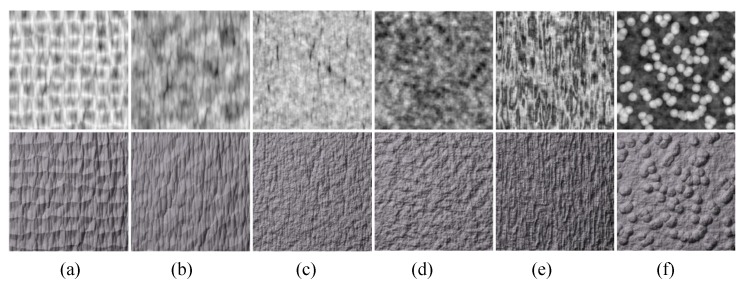
Textures generated with combination methods: (**a**–**c**) are textures generated with a combination of textons and cellular; and (**d**–**f**) are textures generated with combination of textons and Perlin noise. First, row: height maps; Second row: relighting results.

By varying the different parameters, we can generate a large variety of textures, which may be dramatically different from the original textures. Figure 15 shows some textures produced by combination methods.

## 5. Perceptual Properties for Procedural Methods

In this section, we compare different procedural texture generation models and present their strengths and weaknesses. The comparison is based on the appearances of textures that the different models can produce and using the texture dimensions defined by [68]. With an understanding of perception for procedural textures, users will be able to choose procedural models and easily produce a desired perceptual appearance.

### 5.1. Texture Samples

Twenty representative texture generation methods were selected, and each method was used to generate a large number of textures. These methods included Cellular automata, Cellular, Folding (texton, cellular, fractal, and Perlin), Fractal, Combination (cellular/texton, perlin/cellular, and perlin/texton), Islamic pattern, Matrix transformation, Perlin noise, reaction–diffusion, Texton (addition, probability map, randomized grid, random walk, and regular grid), and Wavelet noise. The resolution of each generated height map was 512×512 pixels. For each method, we chose samples that can best represent the possible textures that the method can generate; thus, the number of representative textures for each method was different. For example, the Matrix transformation method had 66 samples (the most) and the random grid Texton method had only seven samples (the least). Overall, we had 450 texture samples. Each sample was rendered under the same area lighting conditions and diffuse reflectance using LuxRender, which is a non-commercial package for realistic graphics rendering. We renamed the filenames of all samples to avoid interfering with the judgment of observers by knowing the generation model in advance. Each rendered texture was printed on 4×4 inch photographic paper with a resolution of 128 pixels per inch for psychophysical experiments.

### 5.2. Subjects

Forty graduate students who had majored in computer science participated in the study, including males and females aged from 23 to 35.

### 5.3. Selection of Perceptual Properties

We used a set of 12 dimensions, based on the texture dimensions defined by [68,69]. The features included contrast, repetition, granularity, randomness, roughness, feature density, directionality, structural complexity, coarseness, regularity, local orientation, and uniformity.

### 5.4. Procedure

Due to the large number of texture samples, it was difficult to display all of them to the observer. Thus, the samples were divided into fifteen groups, with 30 textures in each group. The order of the images in each group was randomized using a Latin Square approach. Every observer was assigned with two or three groups of texture images. The observer needed to go through all the textures in each group and check every image carefully. Then, the observer rated the sample on twelve 9-point Likert scales.

### 5.5. Results

A matrix was constructed by averaging the Likert scale data of each sample. Due to the inconsistent criteria each experimenter used during experiments, the data were normalized, such that they could be compared under the same criteria. For this, the min-max normalization method was used. Supposing that min and max are the minimum and maximum values of the set, respectively, the min-max normalization method maps a value *x* in a set to $x^{\prime }$ in the range of [0, 1] by computing $x^{\prime }=\left(x-min\right)/\left(max-min\right)$.

For each texture generation method, a characteristic description was quantitatively defined by using the average of the normalized Likert scale of all samples generated by the method. It should be noted that this can only be seen as a rough guide for general use, as the perceptual features of different textures generated by the same method could be slightly different.

A 20×12 matrix was obtained through the procedure mentioned above. The row of the matrix represented different texture generation methods and the column represented the texture dimensions. In order to clearly understand the characteristics of each generation method, we transformed the matrix to a 5-point Likert scale and obtained the corresponding semantic description.

We believe that scale 3 represents ’medium’ or ’no obvious’, which means that, for a certain perceptual feature (e.g., coarseness), texture samples generated by a method do not commonly have this feature; that is, the quantitative scales of this feature across different samples generated by the same method were varied, or this perceptual feature was not obvious.

**Figure 16:**
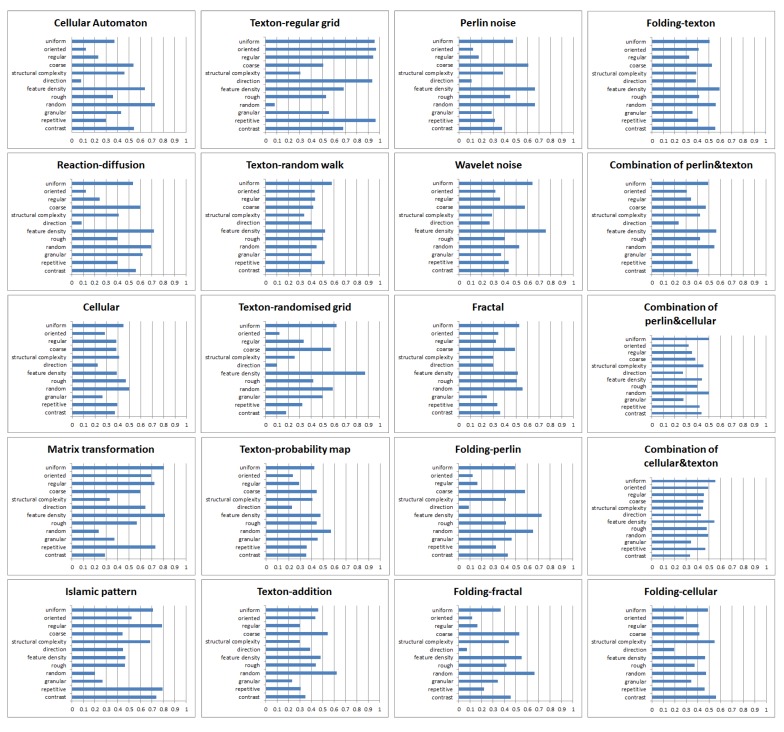
Feature histograms of different texture generation methods.

For the numbers 1,2,4, and 5, we believe that texture samples generated by a certain method shared this common feature and the strength of this feature could be easily judged. Figure 16 shows the feature histograms of different texture generation methods.

### 5.6. Comparison of Procedural Models

Based on the previous sections, we present a detailed comparison of procedural methods for two-dimensional texture generation. We show the results of the comparison in Table 1, in which we compare the nine methods analyzed in this section (except for post-processing methods). In the table, we compare the methods in terms of parameters, perceptual properties, and typical applications. In all of our experiments, based on a desktop computer, all the methods we compared were highly efficient, with computation generally being within one second for generating one texture image. Thus, no computational complexity values are provided in this table.

The column “Parameters” in Table 1 lists the main parameters for each method. Note that there are typically several approaches belonging to one method, and the parameters for each approach are different. Therefore, we only listed the parameters of a typical approach for each procedural method.

The column “Common perceptual properties” in Table 1 lists the perceptual properties shared by textures generated by each method, while the column “Variation of perceptual properties” lists properties which vary between the texture samples generated by an individual method. We compare the quantified average Likert scales and the standard deviation for each perceptual property of textures produced by one model. A small variance means that the textures produced by a certain model has a small change in such perceptual features. Perceptual features with small variance and high average scales are, thus, listed as common perceptual properties. A large variance means that a certain model can produce textures with a large range of variation.

The “Application” column lists the use of procedural methods, in relation to natural surfaces they can simulate.

We conclude from our comparison that every procedural method generates a specific type of texture, and the most suitable method for a specific application depends on the requirements of the particular desired surface.

## 6. Conclusions

We have presented a detailed survey of two-dimensional procedural texture generation methods, dividing these methods into two categories: structured texture and unstructured texture generation methods. We have also discussed filtering and post-processing methods.

Structured textures contain more phase information, which contributes to their structural appearance. Structured texture generation methods include cellular automata, texton placement, reaction–diffusion models, and geometry-based approaches, including Cellular, Voronoi Diagrams, Islamic Patterns, and Matrix transformation methods.

Unstructured textures may be contrary: changing phase information in this type of texture does not necessarily change the appearance of the texture. Unstructured texture generation methods include frequency domain methods, such as colored noise, wavelet noise, anisotropic noise, Fourier spectral texture, spot noise, Gabor noise, and stochastic subdivision functions, or spatial domain methods, such as lattice gradient noise, physically–based simulation, and better gradient noise methods.

Filtering and post-processing methods may take various textures as input and create new textures by changing the different parameters used in the set of filters. In addition, different procedural texture models may be combined to generate various new textures.

We further analyzed the existing procedural texture generation methods and presented a taxonomy of these methods, mainly based on their mathematical foundations. Our conclusions were that reaction–diffusion and cellular automata methods belong to the same class, as they are closely connected mathematically; texton placement and matrix transformation methods can be classified into the same category as their basic operating rule is similar; Voronoi texture, cellular texture, and Islamic pattern generation methods also share some common features, as they are based on Thiessen polygons, which are polygons whose boundaries define the area that is closest to each point relative to all other points.

Finally, we designed a psychophysical experiment to identify the perceptual features of representative textures. An analysis of the results illustrated the strengths and weaknesses of these methods.

It should be noted that each procedural texture generation model can generate a large number of texture samples, and we cannot possibly list all such samples in this survey paper, due to limited space. Therefore, we only selected the most representative samples and presented them in the figures. Although we used 450 samples, a larger number of textures is always preferable.

There are several challenging directions for future work. Firstly, example-based procedural texture generation is very demanding for interactive applications. Procedural texture generation is mathematically random, in terms of content based on given parameters. Creating desirable procedural textures is a time-consuming process of selecting a combination of procedures and parameters. Moreover, the parameters of one model will produce overlapping effects on the output texture appearance and, accordingly, it is difficult to evaluate the perceptual influence of each parameter on the output texture. Thus, to find appropriate procedural texture models and parameter sets to fit a user-specified texture exemplar is an interesting direction for future work. Secondly, natural textures may be edited by procedural methods. Manually editing images in a pixel-by-pixel fashion is complicated and time-consuming. Although some powerful texture generators (e.g., Allegorithmic Substance Designer) exist, they cannot make modifications directly to the intrinsic perceptual attributes of natural textures. If we can change the appearance of a natural texture procedurally—more generally speaking, representing the natural texture in a procedural manner and editing the appearance of the texture by only tuning a few parameters—we may be able to yield high-quality textures in a surprisingly efficient way, based on user requirements. We hope our work can inspire more studies into new methods for procedural texture representation and editing.

## Figures and Tables

**Table 1:** Comparison of perceptual properties of procedural models.

Procedural Models	Parameters	Common Perceptual Features	Variation in Perceptual Features	Applications
Cellular Automaton	finite space, initial state, rules of evolution	non-directional, granular, structural complexity, irregular	structural complexity, uniform, rough	Turing pattern, stripes and spots
Cellular	distance, basis functions	non-directional, coarse, non-oriented	non-granular, rough, feature density, structural complexity, uniform	Sponge, lizard scales, pebbles, flagstones
Fractal	basis function, fractal increment parameter, number of frequencies	non-directional, coarse, irregular, non-oriented	granular, uniform, feature density	terrain, clouds, mountain ranges, coastlines, snowflakes
Islamic Pattern	regular n-gon polygons, n-fold star, rosette, or extended rosette	repetitive, regular, uniform	granular, feature density	carpets, ceramics, leather, stained glass, woodwork, wallpaper
Matrix Transformation	source image, transformation matrix	repetitive, regular, uniform, feature density, directional, locally oriented	granular, feature density, structural complexity	fabric, woven cloth
Perlin Noise	weights for spectral control	non-granular, random, non-directional, irregular, non-oriented	granular, rough	fire, smoke, clouds
reaction–diffusion	chemicals, diffuse rates	random, non-directional, irregular, non-oriented	granular, feature density, structural complexity	spots, stripes, biological
Texton	regular grid, geometric ellipsoid, placement rule	repetitive, non-random, directional, locally oriented, uniform, feature density	structural complexity, granular	weave, leather, Bark
Wavelet noise	weights for spectral control	non-granular, random, non-directional, irregular, non-oriented	granular, rough	fire, smoke, clouds
